# Protumoral TSP50 Regulates Macrophage Activities and Polarization via Production of TNF-α and IL-1β, and Activation of the NF-κB Signaling Pathway

**DOI:** 10.1371/journal.pone.0145095

**Published:** 2015-12-18

**Authors:** Cheng Yang, Dong-Mei Zhang, Zhen-Bo Song, Ya-Qin Hou, Yong-Li Bao, Lu-Guo Sun, Chun-Lei Yu, Yu-Xin Li

**Affiliations:** 1 National Engineering Laboratory for Druggable Gene and Protein Screening, Northeast Normal University, Changchun 130024, China; 2 Institute of Genetics and Cytology, Northeast Normal University, Changchun 130024, China; University of Kansas Medical Center, UNITED STATES

## Abstract

Testes-specific protease 50 (TSP50) is abnormally overexpressed in many kinds of cancers and promotes cell proliferation and migration. However, whether TSP50 can influence the tumor microenvironment, especially the function of immune cells in the microenvironment, remains largely unknown. We demonstrated that exposure to the conditioned medium from TSP50-overexpressing cells, or co-culture with TSP50-overexpressing cells, enhanced the cytokine production and phagocytic activities of macrophages, and induced M2b polarization. Further investigation showed that production of TNF-α and IL-1β was strongly induced by TSP50 in TSP50-overexpressing cells. TSP50-induced TNF-α and IL-1β were main factors that mediated the effects of TSP50-overexpressing cells on macrophages. The NF-κB pathway could be activated in macrophages upon the treatment of conditioned medium of TSP50-overexpressing cells and its activation is necessary for the observed effects on macrophages. Taken together, our results suggested that oncogenic TSP50 expressed in cells could activate surrounding macrophages and induce M2b polarization, partly through inducing TNF-α/ IL-1β secretion and subsequent NF-κB pathway activation. This implies a potential mechanism by which oncogene TSP50 regulates tumor microenvironment to support tumor development.

## Introduction

Oncogenes are required for tumorigenesis, and for continuous tumor cell growth and survival [1]. Testes specific protease 50 (TSP50), a member of the peptidase S1 family of serine proteases, was demonstrated to be oncogenic in previous studies. TSP50 was discovered in breast cancer cells and is normally expressed in human testes but is hardly detected in other tissues [2, 3]. However, in many cancer tissues, TSP50 is abnormally highly expressed [4]. High levels of TSP50 are related to poor prognosis in colorectal carcinoma and gastric cancer patients [5, 6]. Overexpression of TSP50 could promote cell proliferation, migration, and the ability of anchor-independent colony formation via activation of the NF-kB signaling pathway [7–9], whereas downregulation of TSP50 repressed cell proliferation and induced apoptosis [9, 10]. Moreover, the threonine catalytic site of TSP50 is essential for its cell proliferation-promoting activity [8].

In recent decades, it has been widely accepted that cancer development is closely related to the interactions between cancer cells and their surrounding microenvironment [11]. The complex interactions among the various cell types in the tumor microenvironment are understood as an emerging hallmark of cancer [12]. The cellular constituents in the tumor microenvironment are complicated. In addition to neoplastic cells, tumors comprise stromal cells, neovasculature and immune infiltrates. Infiltrating immune cells are increasingly accepted to be generic constituents of tumors. Dysregulation of immune cells and aberrant changes of inflammatory molecules make tumor cells develop the ability to avoid immune destruction [12]. In addition, malignant cells also influence the host microenvironment to facilitate tumor development. Our previous studies elucidated the role of TSP50 in inducing cell transformation and proliferation [7, 8, 10]. However, whether TSP50 has any effect on the tumor microenvironment has not been reported.

Immune infiltrates in the tumor microenvironment include lymphocytes, macrophages, natural killers (NK), dendritic cells (DC), eosinophils, mast cells and myeloid-derived suppressor cells (MDSC)[12]. Among these cells, macrophages are the most abundant innate immune cells [2, 13]. Macrophages are involved in removing apoptotic cells and cellular debris, and also function to regulate homeostasis. The original hypotheses proposed that macrophages are involved in anti-tumor immunity. However, there is substantial clinical and experimental evidence that in the majority of cases, tumor-associated macrophages (TAMs) enhance tumor progression to malignancy. Accordingly, the increasing density of macrophages in cancers is strongly correlated with poor patient prognosis [14]. Macrophages are highly plastic cells and rapidly change their physiology in response to different environmental stimuli [15]. Macrophages can differentiate into two distinct phenotypes: the classically activated (M1) macrophages and the alternatively activated (M2) macrophages, which promote Th1 and Th2 responses respectively [16, 17]. M1 macrophages are stimulated by IFN-γ, lipopolysaccharide (LPS) or tumor necrosis factor-a (TNF-a) and secret a series of pro-inflammatory cytokines, chemokines and effector molecules, such as TNF-α, IL-1β, IL-6, IL-12, IL-23 and iNOS [16, 18]. M2 macrophages are polarized by distinct stimuli and are further subdivided into M2a, M2b and M2c macrophages. M2a macrophages are stimulated by the Th2 cytokines, IL-4 or IL-13, and M2b macrophages are induced by immune complexes, LPS, Toll-like receptors (TLRs) or IL-1. Finally, M2c macrophages are induced by IL-10, transforming growth factor-b (TGF-β) or glucocorticoids. Overall, M2 macrophages express a high level of anti-inflammatory cytokine IL-10, but low levels of IL-12 and IL-23, although different M2 subtypes may secrete certain cytokines differently. Moreover, differentially polarized macrophages also express distinct chemokines such as chemokine (C-X-C motif) ligands (CXCLs) and chemokine (C-C motif) ligands (CCLs), which recruit different immune cells and drive different immune responses[19]. Therefore, M1 and M2 macrophages have different properties and play distinct roles during tumor development. However, how malignant cells influence the function and polarization of the surrounding macrophages remains largely unknown.

To explore the influence of oncogene TSP50 on the surrounding immune environment of tumors, we established a TSP50-overexpressing cell line (TSP50-o/e cells) and studied the effects of the conditioned medium (CM) from TSP50-o/e cells on macrophages. Our results demonstrated that exposure to the CM of TSP50-o/e cells enhanced cytokine production and phagocytic activities of macrophages, and induced M2b polarization. Furthermore, we identified TNF-α and IL-1β, which were highly induced by TSP50 in TSP50-o/e cells, as important effectors that mediate the effects of the CM of TSP50-o/e cells on macrophages by activating the NF-κB pathway. Overall, our results suggest a potential mechanism by which the oncogene TSP50 is involved in regulating tumor microenvironment to support tumor development.

## Materials and Methods

### Ethical Treatment of Animals

C57BL/6 mice and BALB/c nude mice were purchased from the Experimental Animal Center Jilin University (Changchun, China). The animal experiments were carried out in strict accordance with the recommendations in the Guide for the Care and Use of Laboratory Animals of the National Institutes of Health. The Chinese Academy of Sciences Animal Care and Use Committee gave approval for the animal experiments. All surgery was performed under sodium pentobarbital anesthesia, and all efforts were made to minimize suffering.

### Antibodies and Reagents

Polyclonal antibodies against p65 (D1513), IκBα (F0613), anti-IKKα/β antibodies (H2208), and goat antibody against Histone H1 (A2713) were purchased from Santa Cruz Biotechnology (Santa Cruz, CA, USA). Anti-Phosphors-IκBα (9246), anti-phosphor-IKKα/β (2697), and anti-Phosphors-p65-Ser536 (3033) were obtained from Cell Signaling (Beverly, MA, USA). Anti-CCL1 (AF272 and AF845) (1:1000), anti-CCL5 (AF278 and AF478), human recombinant TNF-α, and human recombinant IL-1β were obtained from R&D systems (Boston, MA, USA). The monoclonal antibody against TSP50 was generated in our laboratory. The mouse monoclonal antibody against GAPDH (1504) was purchased from Kangcheng Biotech (Shanghai, China). The above primary antibodies were 1 to 1000 diluted when used for Western blot, except that anti-Histone H1 antibody was used with 1 to 500 dilution. APC anti-mouse CD11b (M10118) was from Sungene Biotech (Tianjin, China) and the anti-mouse F4/80 antigen FITC (02922–50) was from Biogems (Westlake Village, CA, USA). HRP-conjugated goat anti-mouse antibodies (IH-0031), HRP-conjugated goat anti-rabbit antibodies (IH-0011), and HRP-conjugated horse anti-goat (IH-0051) were purchased from Dingguo Changsheng Biotechnology (Beijing, China) and 1 to 2000 diluted when used for Western blotting. Phorbol myristate acetate (PMA) and the total nitric oxide assay kit was purchased from Beyotime Institute of Biotechnology (Shanghai, China). Ammonium pyrrolidine dithiocarbamate (PDTC) and N-acetylcysteine (NAC) were obtained from Sigma Aldrich (St Louis, MO, USA).

### Cell Culture

Chinese hamster ovary cells (CHO), THP-1 cells and RAW264.7 cells were obtained from the Cell Bank of the Chinese Academy of Sciences (Shanghai, China). THP-1 cells were induced to differentiate (dTHP-1) upon exposure to 0.2μg/mL PMA in the culture medium for 24h. Mouse peritoneal macrophages were obtained from ascites of C57BL/6J mice that were injected intraperitoneally with 1mL of 5% soluble starch 3 days before sacrifice.

CHO cells, mouse peritoneal macrophages and RAW264.7 cells were cultured and propagated in Dulbecco’s modified Eagle’s medium (DMEM) (Gibco, Grand Island, NY, USA), supplemented with 10% fetal bovine serum (Every Green, Hangzhou, China), 100 U/mL penicillin and 100 mg/mL streptomycin (Ameresco, Solon, OH USA). dTHP-1 cells were cultured in RPMI 1640 medium (Hyclone, Logan, UT, USA) supplemented with 10% fetal calf serum, 0.7μL/L beta-mercaptoethanol and 2.235g/L Hepes. The cells were cultured at 37°C in a humidified atmosphere with 5% CO_2_ and 95% air.

### Stable Transfection

The pcDNA3.0-TSP50 expression plasmid has been described previously [8]. CHO cells were transfected with pcDNA3.0-TSP50 and pcDNA3.0 empty plasmids (negative control), separately, using Lipofectamine TM2000 (Invitrogen, Grand Island, NY, USA). The cells were then cultured with selection-medium (G418 400μg/mL) from the second day of transfection for 2 weeks to select stably transfected cells. The selected positive cells were cultured under the G418 selection-medium for 2 months, and then maintained in the culture medium containing 200μg/ml G418. TSP50 expression in the stably transfected cells was verified by western blotting. The cell line transfected with pcDNA3.0-TSP50 was designated TSP50-o/e cells [20].

### CM preparation

To prepare the CM, TSP50-o/e cells and control cells were seeded at a density of 1×10^6^ cells/mL and cultured in complete medium for 24 h. Subsequently, the cells were shifted to fresh medium and cultured for another 24 h, after which the CM was harvested, centrifuged and stored at −80°C before use.

### Preparation of shRNA and siRNA

To create the TNF-α shRNA expression vector, the pRNAT-U6.1/Hygro vector (GenScript Corporation, IA, USA) was used and manipulated as previously described [8]. The two shRNA sequences targeting TNF-α were TNF-α shRNA#1 5′-GGCAGTCAGATCATCTTCT-3′ and TNF-α shRNA#2 5′-GACAACCAACTAGTGGTGC-3′. The sequence of the negative control shRNA (GenScript Corporation, China) was 5′-GACGCTTACCGATTCAGAA-3′, which has no significant homology to mouse or human gene sequences. The scrambled sequences of TNF-α shRNA were S-shRNA#1 5′-GAGGTACACACATCGATCG-3′ and S-shRNA#2 5′-GAGACTACAGGACTACGTC-3′. TSP50-o/e cells and control cells (1×10^6^) were seeded into each well of 6-well plates and transfected with 2μg shRNA using Lipofectamine TM2000 (Invitrogen). Cells were cultured for another 24 h before the CMs were harvested.

The siRNA study included two mouse siRNAs designed against IL-1β, two scrambled siRNAs and one negative control siRNA (GenePharma, Shanghai, China). The sequences for control siRNAs and IL-1β siRNAs were as follows: negative control siRNA (5′-UUCUCCGAACGUGUCACGUTT-3′), IL-1β siRNA No.1 (5′-GUGGUCAGGACAUAAUUGATT-3′),GAPDH positive control (sense: 5′-GUAUGACAACAGCCUCAAGTT-3′), IL-1β siRNA No.2 (5′-CCCAAGCAAUACCCAAAGATT-3′), the scrambled sequences of related IL-1β siRNA No.1 (5′- GAUUGAUAAUUGCGGAGACTT-3′) and the scrambled sequences of related IL-1β siRNA No.2 (5′-AGAACCAACCGAAUACCACTT-3′). Each freeze-dried siRNA was reconstituted with RNase-free water to prepare a 20 μM stock solution [21]. Before transfection, TSP50-o/e cells and control cells (1×10^6^) were seeded into each well of 6-well plates with completed growth medium for 24 hours. Cells were incubated at 37°C with 5% CO_2_ overnight and treated with 150 pmol siRNA per-well. The siRNAs were incubated with the Lipofectamine 2000 Transfection Reagent (CA, USA), according to the manufacturer’s instructions. After incubation for 12 hours, cells were washed twice with phosphate-buffered saline (PBS), and the medium was replaced by fresh medium with 3% FBS. Subsequently, cells were cultured for another 24 h before the CM was harvested, centrifuged and stored at −80°C before use.

### RNA Extraction and Real-time PCR

Before being subjected to RNA extraction, macrophages were incubated in medium containing 30% CM from TSP50-o/e cells or control cells for 24 hours. Total RNA was extracted according to the manufacturers’ instructions (Invitrogen). 50 ng of cDNA was subjected to real-time PCR in a final volume of 10 μL, containing one set of primers and the SYBR Green I PCR master mix (TAKARA, Dalian, China). The amplification was carried out with an ABI thermocycler (Applied Biosystems) as follows: 95°C for 1 min and then 40 cycles of 95°C for 5s and 60°C for 1min. Relative expression of each gene was calculated using the ΔΔCT method. The data was expressed as the relative expression of the target genes between the experimental group and the control group. All real-time PCR reactions were performed in triplicate.

### Western Blotting Analysis

Cells were extracted using lysis buffer (0.5% Nonidet P-40, 20 mM Hepes-NaOH (pH 7.9), 20 mM NaF, 1 mM EDTA, 1 mM DTT, 0.4 mM phenylmethysulfonyl fluoride, 2 μg/ml leupeptin, 2 μg/ml pepstatin and 2 μg/ml aprotinin) with occasional rocking for 30 min, and then centrifuged at 12,000×g, for 10 min at 4°C. The supernatant contained the cytosolic extracts. The precipitant was lysed with a lysis buffer (420 mM NaCl, 20% glycerol, 20 mM Hepes-NaOH (pH 7.9), 20 mM NaF, 1 mM EDTA, 1 mM DTT, 0.4 mM phenylmethysulfonyl fluoride, 2 μg/ml leupeptin, 2 μg/ml pepstatin and 2 μg/ml aprotinin) for nuclear extracts. Equal amounts of protein were resolved by 12% sodium dodecyl sulfate-polyacrylamide gel electrophoresis (SDS–PAGE). The proteins were transferred to polyvinylidene difluoride (PVDF) membrane, and blocked with 5% non-fat dry milk in TBST (20 mM Tris-HCl (pH 7.6), 150 mM NaCl, and 0.02% Tween20) at room temperature for 1 h. The membranes were sequentially reprobed with primary antibodies overnight and HRP-conjugated secondary antibodies for 30 minutes at room temperature. The signals were detected with sECL reagent (Beyotime,) [8]. Results were quantified using ImageJ.

### Phagocytosis assays with RBCs and Yeast

To evaluate the phagocytic capability of mouse macrophages, chicken red blood cells (cRBC) and yeast cells were used as antigen particles. Mice were euthanized by cervical dislocation, and 2 ml of saline was injected into the abdominal cavity to collected peritoneal macrophages. Macrophages were plated in 6-well plate at 1×10^5^/well in 1 mL of 10%-FBS DMEM and cultured overnight. Then, 0.5 mL of the CM was added into each well to treat the macrophages for another 24h. Macrophages were incubated in 10%-FBS DMEM containing 0.5% cRBCs or 10^8^/mL yeast-eosin solution for 2h.The number of macrophages that ingesting cRBC out of a total of 200 cells was calculated by direct visual counting under a light microscope. The phagocytosis index of macrophages were calculated as follows: phagocytosis index (PI) = the number of macrophages phagocytosing cRBC or yeast cells /200×100% [22].

### Transwell chamber co-culture

Mouse peritoneal macrophages or dTHP-1 cells were loaded onto a 24-well plate at a density of 1×10^5^/well (lower chamber). The 0.4μm pore-size transwell insert (Coring, USA) was then placed into each well and 0.5×10^5^ of TSP50-o/e cells or control cells in 0.2 mL of DMEM were added onto the filter of each insert. Cells were cultured for 24h before analysis.

### ELISA

Equal volumes of cell culture supernatants were collected and stored at −80°C before use. Then the levels of human IL-1β, IL-10, IL-12, murine IL-1β, IL-6, IL-10, IL-12, TNF-α or TGF-β in the supernatants were determined using commercially available ELISA kits (R&D Systems, Minneapolis, MN, USA), following the protocols supplied by the manufacturers.

### Protein Precipitation

One volume of 100% (W/V) Trichloroacetic Acid (TCA) stock was added into four volumes of protein sample and incubated for 1 hour at 4°C, before being centrifuged at 12000rpm for 45 min. The supernatant was removed and the protein pellet was washed with 0.2mL cold acetone twice, and centrifuged at 12000rpm for 45 min. The pellet was dried at room temperature for 30min to drive off the acetone. 20 μLof 4×sample buffer was then added and the samples were boiled for 10 min at 100°C before loading samples onto an SDS-PAGE gel [23].

### Tumorigenicity studies

Six-week old BALB/c nude mice were housed under pathogen-free conditions. TSP50 o/e cells and control cells were grown, harvested and resuspended in PBS at 5×10^5^ cells/mL. 200μl of the cell suspension was dorsally and subcutaneously injected into nude mice [7]. The tumor size was determined using calipers. The animal experiments were performed in accordance with established guidelines.

### Tumor digestion and FACS analysis

TSP50-o/e and control tumors were excised, minced and digested to single cell population before being purified using the magnetic-activated cell sorting (MACS) method (Militenyi Biotec, Bergisch Gladbach, Germany), according to the manufacturer’s instructions. For fluorescence activated cell sorting (FACS) analysis, cells were incubated with antibodies for 30 minutes. The concentration of these antibodies was 0.25μg/10^6^ cells in 100μl volume. CD11b and F4/80 positive cells were sorted by BD FACSAria II (BD, USA) and used for real time PCR studies.

### Statistical analysis

Experimental data was processed using SPSS statistical software (18.0). Data are expressed as means ±SD. Statistical analysis of the data was performed using the Student’s t test. P-values <0.05 were considered statistically significant.

## Results

### 1. The CM of TSP50-o/e cells stimulated the production of Inflammatory Cytokines in Macrophages

**Fig 1:**
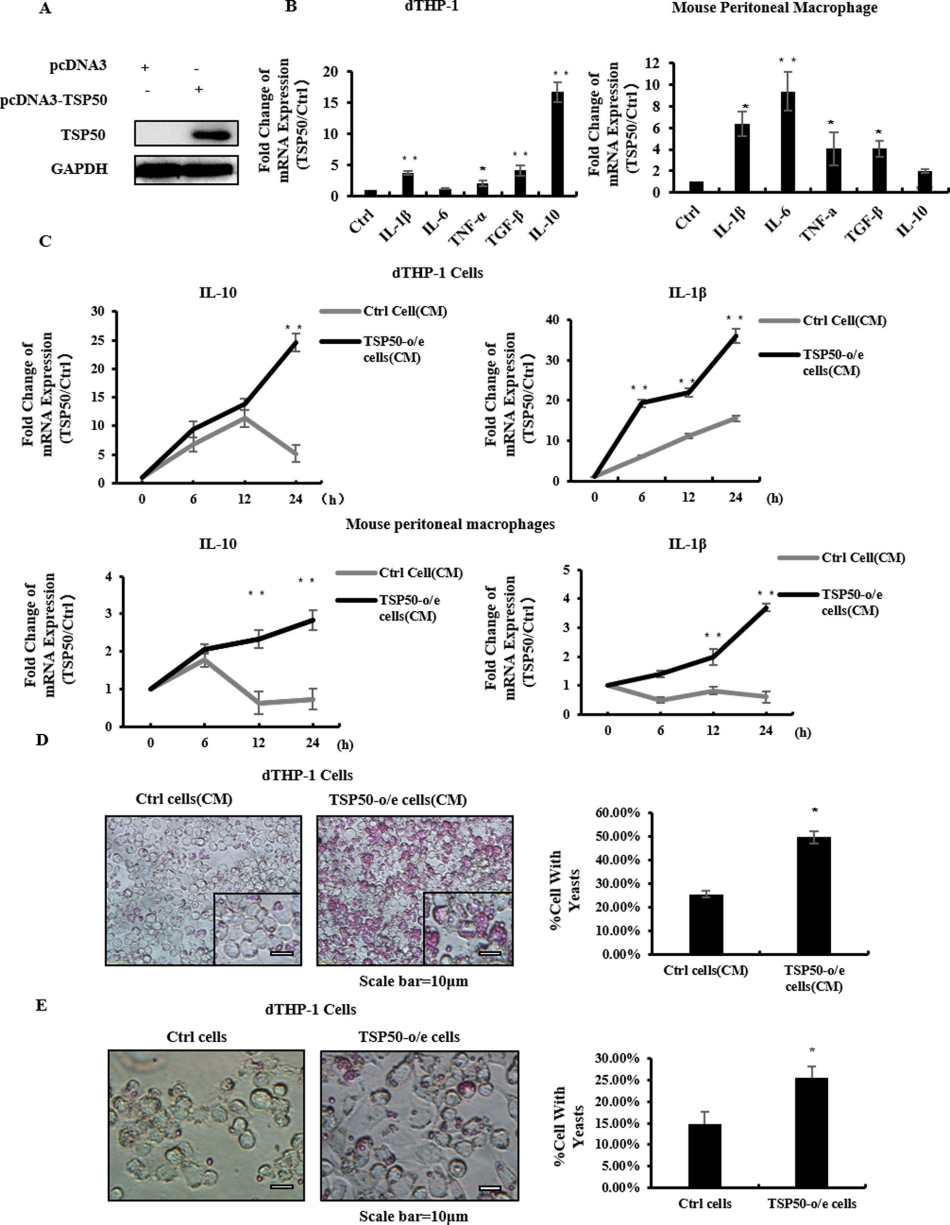
CM of TSP50-o/e cells stimulated the production of inflammatory cytokines and activated phagocytosis in macrophages. (A) Western blot analysis confirming the expression of TSP50 in TSP50-o/e cells. (B) Macrophages were cultured in medium containing 30% of conditioned medium (CM) from TSP50-o/e cells or control cells for 24h. Cytokine production in dTHP-1 cells (left) and mouse peritoneal macrophages (right) were determined by real-time PCR. Results are expressed as the difference between the expressions of the target gene in TSP50-o/e cells relative to the control group. (C) Macrophages were exposed to CM from TSP50-o/e cells or control cells for 6h, 12h and 24h. Cytokine production in dTHP-1 cells (up) and mouse peritoneal macrophages (down) were determined by real-time PCR. Control cells were CHO cells transfected with empty vectors. (D, E) Phagocytosis by dTHP-1 of yeast cells was evaluated after 24 hours of CM treatment (D) or co-culture with TSP50-o/e cells or control cells (E). dTHP-1 cells phagocytizing yeast cells were observed by light microscopy (left) and the calculated phagocytic index is shown (right). GAPDH was used as the internal control to check the efficiency of cDNA synthesis and PCR amplification. Representative results are from one of three independent experiments with similar results. Data are shown as mean ± SD of three replicates in three independent experiments.* p<0.05, **p<0.01.

To test whether oncogene TSP50 facilitates tumor growth through regulating tumor microenvironment, we first established a cell line that overexpressed human TSP50 protein (TSP50-o/e cells). We then investigated the effect of the CM from TSP50-o/e cells on macrophages. CHO cells, in which endogenous TSP50 is hardly detectable, were stably transfected with pcDNA3-TSP50 or pcDNA3.0 empty (negative control) plasmids through G418 selection. Western blot analysis confirmed the overexpression of TSP50 in the stably transfected CHO cells (Fig 1A). The CM of TSP50-o/e cells were then collected and applied onto dTHP-1 cells, mouse peritoneal macrophages or RAW264.7 macrophages for 24h. Real-time PCR analysis showed that following the treatments, the production of representative cytokines was increased to different degrees: the expressions of *IL-1β*, *TNF-α* and *IL-10* significantly were increased in the three types of macrophages (Fig 1B and S1A Fig). We then exposed macrophages to the CMs and harvested them at 6h, 12h and 24h. Real-time PCR analysis revealed that the mRNA expressions of *IL-10*, *IL-1β*, *IL-6* and *TNF-α* were increased at each point compared with the control group (Fig 1C and S1B Fig). These results suggest that the CM from TSP50-o/e cells could stimulate inflammatory cytokines production from both macrophage cell lines and primary macrophages.

### 2. CM of TSP50-o/e cells Enhanced Macrophage Phagocytic Activity

We evaluated the phagocytic capacity of macrophages to engulf chicken blood cells or yeast cells when treated with the CM of TSP50-o/e cells. In dTHP-1 cells, the phagocytic index was increased by approximately 23% upon treatment with the CM of TSP50-o/e cells compared with that of control CM (Fig 1D). Similarly, mouse peritoneal macrophages exposed to CM of TSP50-o/e cells ingested approximately 15% more yeast cells than those exposed to the control CM (S1C Fig). We then performed co-culture experiments using transwell chambers to mimic the tumor microenvironment. Macrophages were seeded in the bottom chamber of transwell while TSP50-o/e cells or the negative control cells were introduced onto the membrane in the upper chamber. Both human and murine macrophages showed a greater phagocytosis ability when co-cultured with TSP50-o/e cells (Fig 1D and S1C Fig). However, RAW 264.7 cells hardly ingested any yeasts or RBCs after CM treatment; therefore, we performed the subsequent experiments only with THP-1 cells and mouse peritoneal macrophages. Taken together, the above results suggested that CM from TSP50-o/e cells could enhance the phagocytic capacities of macrophages.

### 3. The CM of TSP50-o/e cells polarized macrophages into M2b phenotype

**Fig 2:**
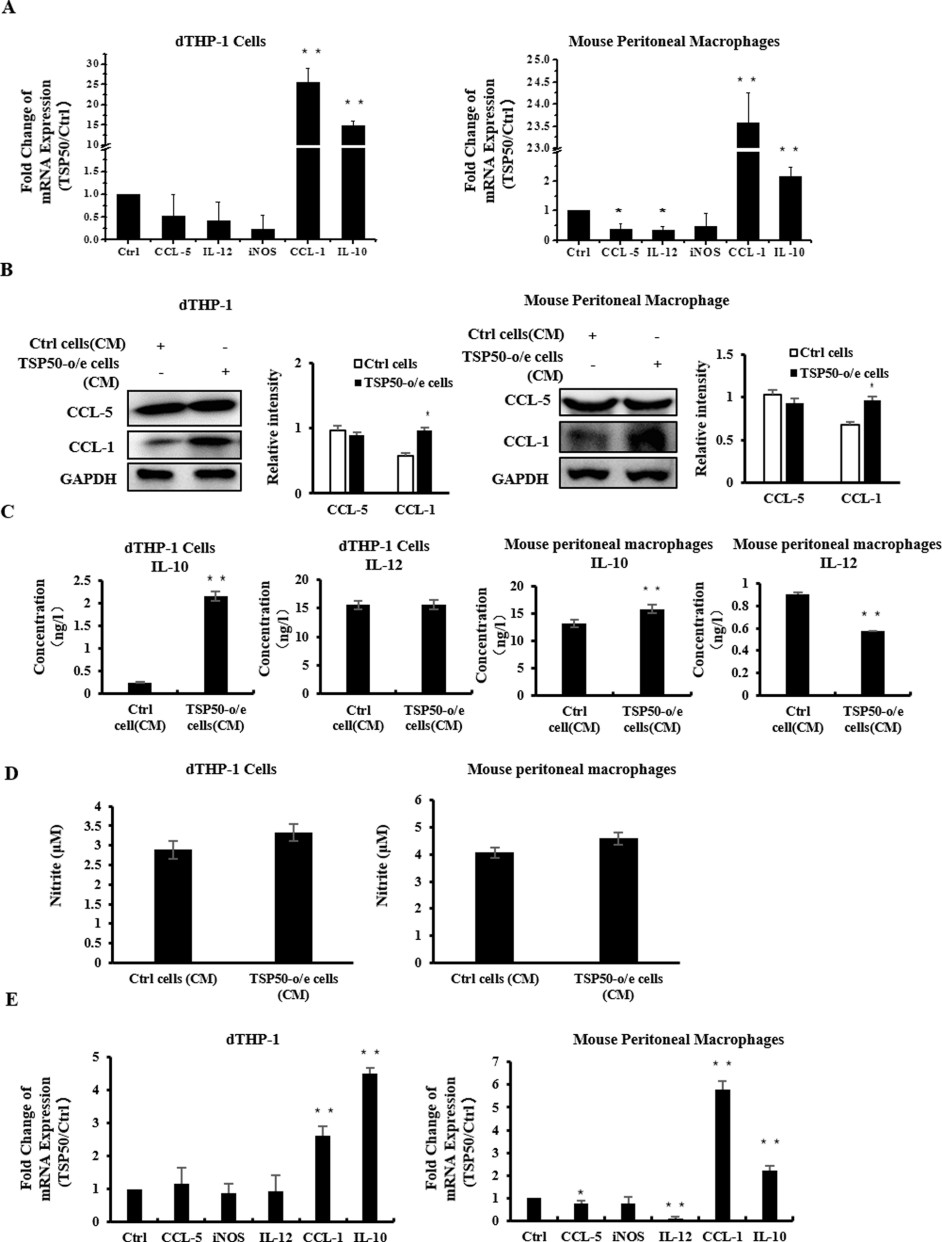
CM of TSP50-o/e cells polarized macrophages to an M2b phenotype. (A) dTHP-1 cells (left) or mouse peritoneal macrophages (right) were treated with 30% of CM from TSP50-o/e cells or control cells for 24h. Expressions of phenotypic markers (CCL-5, IL-12, iNOS, CCL1 and IL-10) were analyzed by real-time PCR. (B) Expressions of CCL5 and CCL1 in dTHP-1 cells (left) and murine macrophages (right) were detected by western blotting. ImageJ was used to quantify the blots. (C-D) dTHP-1 cells or mouse peritoneal macrophages were treated with 30% of CM from TSP50-o/e cells or control cells for 24h. CM from macrophages were collected and subjected to ELISA to detect the secretion of cytokines (C), and nitrite was assayed by the Griess reagent (D). (E) dTHP-1 cells (left) or mouse macrophages (right) were co-cultured with TSP50-o/e cells or control cells for 24h before RNA extraction. Relative mRNA levels of CCL5, IL-12, iNOS, CCL1 and IL-10 in dTHP-1 cells were determined by real-time PCR. GAPDH was used as the internal control for PCR. Representative data from three experiments are shown as mean ± SD. * p<0.05, **p<0.01.

The induced cytokine content (TNF-α^high^, IL-1β^high^ and IL-10^high^) upon exposure to CM of TSP50-o/e cells limited the active macrophages into M1 or M2b phenotypes. To determine the polarized phenotype of the macrophages, we detected the expression of biomarkers for M1 macrophages (CCL-5, IL-12 and iNOS) and M2b (CCL-1 and IL-10) macrophages [13, 17, 19, 24, 25]. The mRNA levels of the M1 marker iNOS and CCL-5 were barely changed in dTHP-1 cells and mouse peritoneal macrophages following exposure to the CM of TSP50-o/e cells (Fig 2A and S1B Fig), and expression of IL-12 was decreased in mouse peritoneal macrophages. The protein level of IL-12 in mouse macrophages was greatly decreased, whereas CCL-5 production did not change (Fig 2B). Comparatively, both the mRNA and protein levels of M2b markers CCL-1 and IL-10 were strongly increased in dTHP-1 and mouse peritoneal macrophages (Fig 2B and 2C). Subsequently, the iNOS level in the CMs was also assayed using Griess reagent, which showed that the level of nitrite, the stable end product of NO was barely changed (Fig 2D). Furthermore, when co-cultured with TSP50-o/e cells, the expression of *CCL-1* and *IL-10* was significantly increased in macrophages, while the expressions of *CCL-5* and *IL-12* decreased (Fig 2E). These results suggested that the CM of TSP50-o/e cells induced macrophages to M2b polarization, and implied the existence crosstalk between TSP50-positive tumor cells and macrophages in the microenvironment.

### 4. TSP50 Promoted Cytokine Production in TSP50-o/e cells

**Fig 3:**
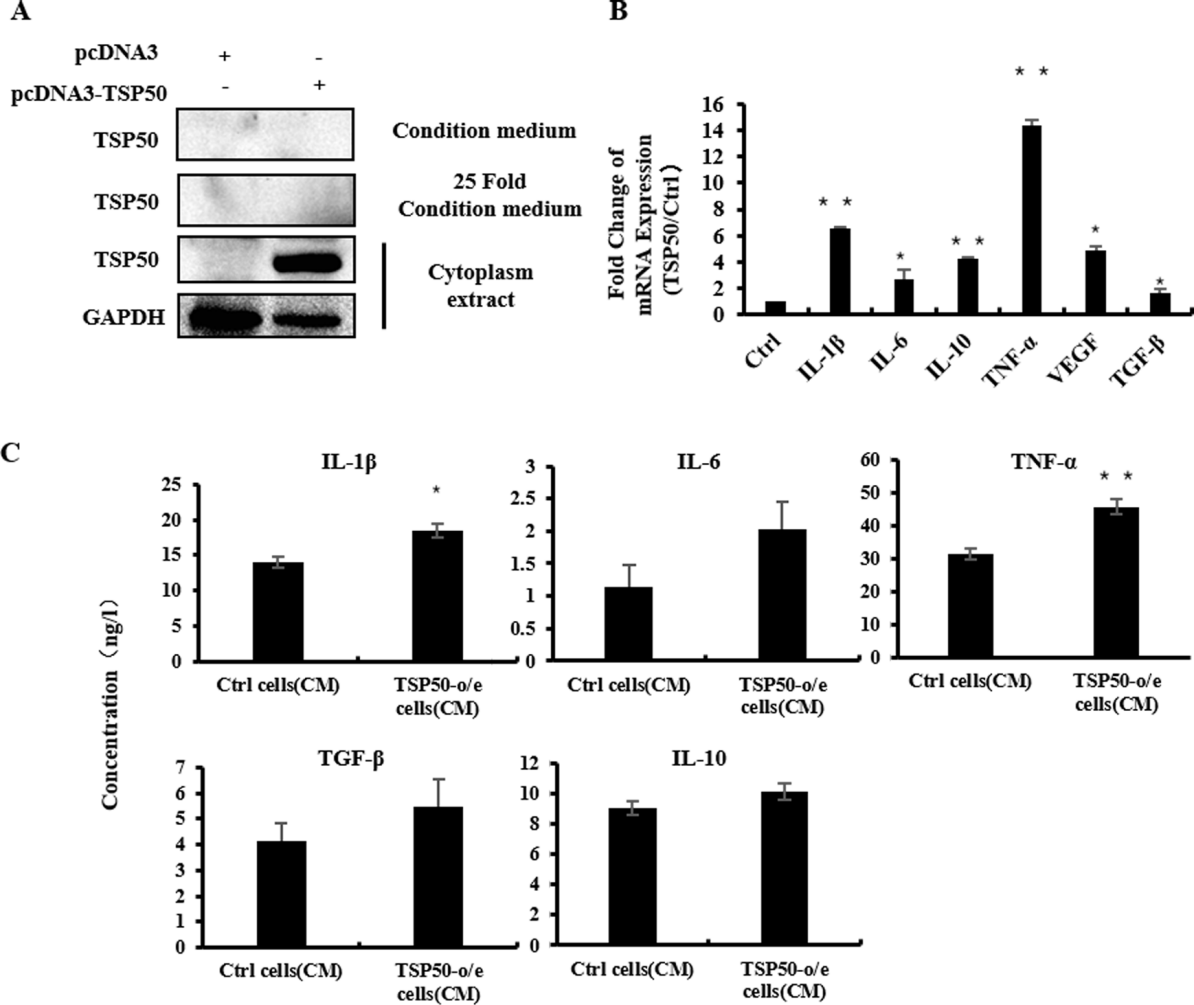
TSP50 promotes cytokine production in TSP50-o/e cells. (A) CM from TSP50-o/e cells and control cells were collected and 25-fold concentrated by trichloroacetic acid (TCA)/acetone precipitation, while cells were lysed to obtain cell lysate. The TSP50 expression in both CM and cells was then detected by western blotting. Results are from one experiment representative of three independent experiments performed with similar results. (B) Expression of cytokines in TSP50-o/e cells was determined by real-time PCR. The fold change relative to control cells is presented. (C) CM from TSP50-o/e cells and control cells were collected and subjected to ELISA to detect the secretion of cytokines. GAPDH was used as the internal control for PCR. Data are shown as mean ± SD of three replicates in three independent experiments.* p<0.05, **p<0.01.

We next determined what components in the CM of TSP50-o/e cells would be responsible for the macrophage polarization effects. We first tested the possibility that TSP50 itself in the CM directly elicited these effects on macrophages. The protein level of TSP50 in the CM was analyzed by western blotting; however, no detectable TSP50 was detected in the CM even after 25-fold enrichment by TCA precipitation (Fig 3A). Oncogene RET/PTC1 has been reported to activate the production of pro-inflammatory cytokines, which in turn contributes to tumor growth and invasion [26]. We then tested whether the same mechanism is involved in the case of TSP50. Real-time PCR analysis showed that the mRNA levels of *IL-1β*, *IL-6*, *TNF-α*, *IL-10*, *VEGF* and *TGF-β* were indeed higher in TSP50-o/e cells than in control cells (Fig 3B). In particular, the mRNA level of *TNF-α* was dramatically increased (by 14.3-fold, p<0.01). The concentration of these cytokines in the CMs was then measured using ELISA (Fig 3C). Consistent with the real-time PCR results, the concentrations of IL-1β, IL-6, TNF-α, IL-10 and TGF-β were increased to various degrees. The concentration of TNF-α in TSP50-o/e cells-CM was increased by 16ng/L compared with that in the control CM (p<0.01) (Fig 3C). Taken together, TNF-α expression increased the most among the measured factors, based real-time PCR and ELISA analyses.

### 5. TNF-α in the CM of TSP50-o/e cells was Crucial for Regulating Macrophage Activities

**Fig 4:**
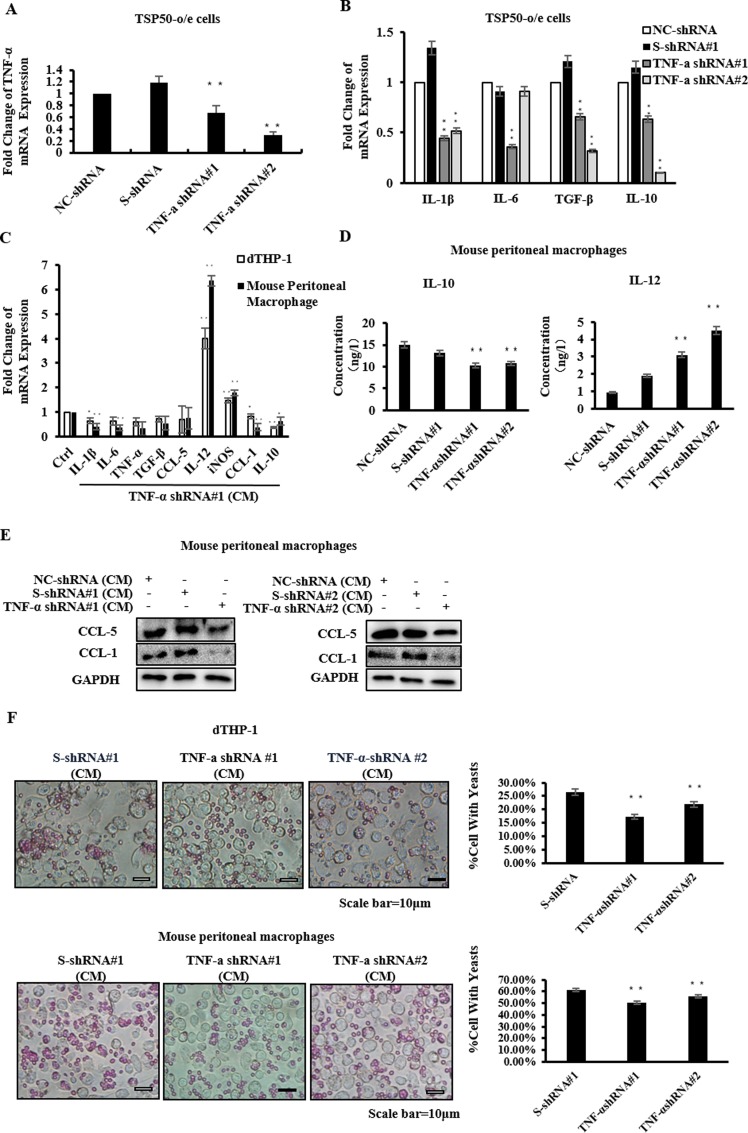
TNF-α induced by TSP50 is crucial for affecting macrophage activities. (A-B) TSP50-o/e cells were transfected with the indicated shRNA plasmids. After 24h, the mRNA level of TNF-α (A) or cytokines (B) in these cells were analyzed by real-time PCR. (C) dTHP-1 cells or mouse peritoneal macrophages were cultured with CM from TNF-α knock-down TSP50-o/e cells or control cells for 24h. The mRNA expression of cytokines and macrophage phenotypic markers were determined by real-time PCR. (D) Mouse peritoneal macrophages were cultured with CM from TNF-α knock-down TSP50-o/e cells or control cells for 24h. CM from macrophages was collected and subjected to ELISA to detect the secretion of cytokines. (E) Mouse macrophages were cultured with CM from TNF-α knock-down TSP50-o/e cells or control cells for 24h. The macrophages were collected and lysed, and the protein level of macrophage phenotype markers were analyzed by western blotting. (F) Phagocytosis capacities of dTHP-1 cells (up) or mouse peritoneal macrophages (down) were measured after treatment with CM from TNF-α knock-down TSP50-o/e cells or control cells. GAPDH was used as the internal control to check the efficiency of cDNA synthesis and PCR amplification. Data are shown as mean ± SD of three replicates in three independent experiments.* p<0.05, **p<0.01.

To validate that TSP50-induced TNF-α production is responsible for the effects of CM from TSP50-o/e cells on macrophages, we first specifically silenced the expression of TNF-α in TSP50-o/e cells using shRNA (Fig 4A and S2A Fig). Following the downregulation of TNF-α, the production of *IL-1β*, *IL-10* and *TGF-β* mRNAs was obviously decreased (Fig 4B). The CM of TSP50-o/e cells with TNF-α shRNA or control shRNA was then collected and added to dTHP-1 cells or mouse peritoneal macrophages. In dTHP-1 cells, the mRNA levels of *IL-6*, *TNF-α*, *TGF-β*, *CCL-5*, *IL-10* and *CCL-1* showed an obvious decrease, whereas the expression of *IL-1β*, *IL-12* and *iNOS* increased (Fig 4C and S2B Fig). In mouse peritoneal macrophages, the mRNA expressions of *CCL-1* and *IL-10* decreased (Fig 4C and S2B Fig), while the expressions of *IL-1β*, *IL-6* and *TNF-*α were increased when exposed to the CM of TNF-α shRNA for 6h, and then declined with ongoing treatment (S2B Fig). Consistent with the real-time PCR results, the production of IL-12 increased while IL-10 expression decreased (Fig 4D). We also validated the changes of CCL-5 and CCL-1 at the protein level (Fig 4E and S2C Fig). Moreover, knockdown of TNF-α abolished the enhanced phagocytic abilities of dTHP-1 cells and mouse macrophages induced by the CM of TSP50-o/e cells (Fig 4F).

Balkwill's group implicated TNF-α in the tumor-macrophage cross-talk[27]. Indeed, when 40ng/mL of TNF-α was added to dTHP-1 cells or mouse peritoneal macrophages, the expression of CCL-1 and IL-1β increased, while CCL-5 expression barely changed (Fig 3A and 3B). Furthermore, addition of TNF-α alone activated phagocytosis of both dTHP-1 cells and mouse macrophages (S3C Fig). The above results indicated that TNF-α, which was induced by TSP50, is a crucial factor that mediates the stimulatory effect of the CM from TSP50-o/e cells on the activities of macrophages.

### 6. IL-1β in the CM of TSP50-o/e cells Affected the Activities of Macrophages

Following the knockdown of IL-1β in TSP50-o/e cells (S4A Fig), the expressions of *TNF-α* and *IL-10* were obviously decreased (S4B Fig). The CMs of TSP50-o/e cells with IL-1β siRNA or control siRNA were collected and applied to macrophages. We observed that mRNA levels of *CCL-1*, *IL-10*, *TNF-α* and *IL-1β* were declined, while *CCL-5* and *IL-12* production were increased (S4C Fig). The protein levels of IL-10 and IL-12 showed a similar tendency (S4D Fig). These results indicated that TSP50-induced IL-1β could also regulate the effect of the CM from TSP50-o/e cells on activated macrophages.

### 7. The CM of TSP50-o/e cells Regulated the Activities of Macrophages via the NF-κB Pathway

**Fig 5:**
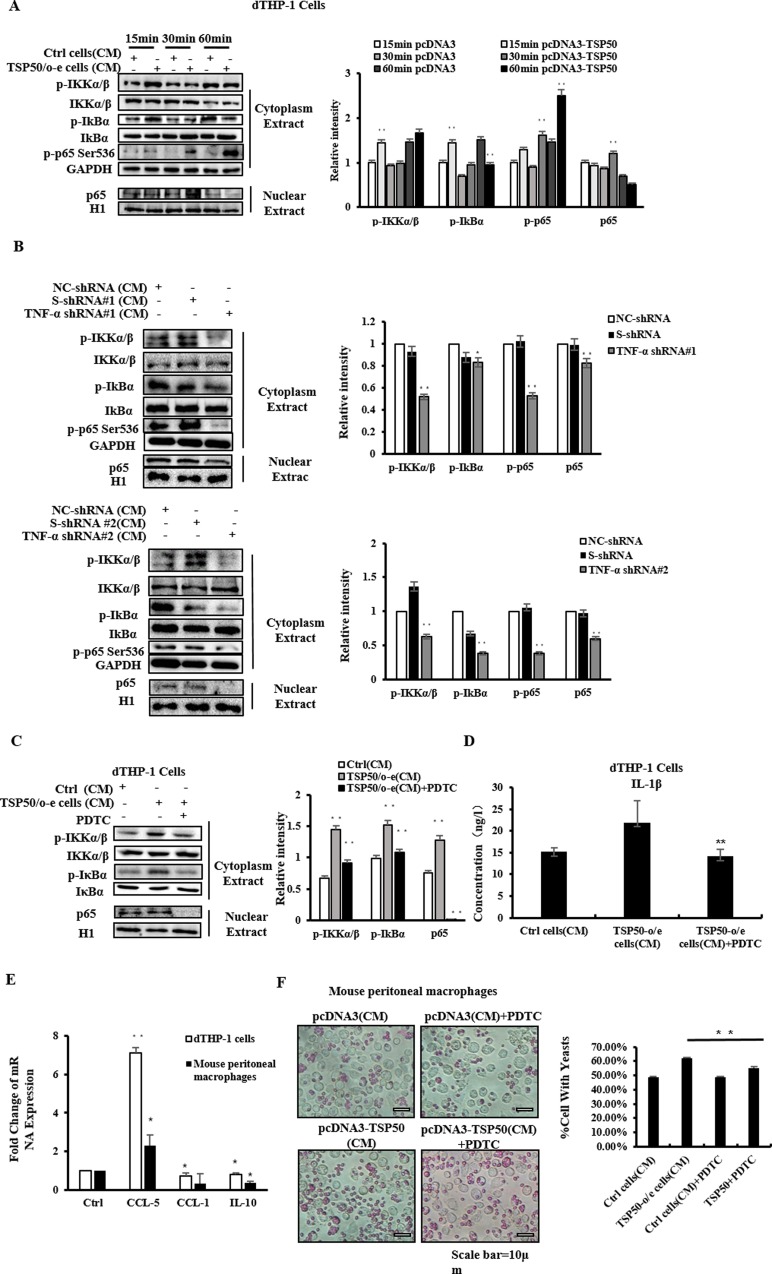
CM of TSP50-o/e cells regulates of macrophages activities via NF-κB pathway. (A) Macrophages were treated with CM from TSP50-o/e cells or control cells for 15min, 30min or 60min. The activation of NF-κB pathway in dTHP-1 cells was then analyzed by western blotting. (B) Mouse macrophages were treated with CM from TNF-α knock-down TSP50-o/e cells for 30min. The activation of NF-κB pathway in dTHP-1 cells was then analyzed by western blotting. (C) dTHP-1 cells were pretreated with 25μg/mL of NF-κB inhibitor PDTC for 30min and then the culture medium were replaced with fresh medium containing 30% of CM from TSP50-o/e cells or control cells. After 30-min incubation, the activation of NF-κB pathway was analyzed by western blotting. (D) dTHP-1 were treated with PDTC and CM from TSP50-o/e cells for 24 hours. The CMs were collected and subjected to ELISA to detect the production of IL-1β. (E) dTHP-1 cells and mouse peritoneal macrophages were incubated in culture medium containing PDTC and CM of TSP50-o/e cells or control cells for 24 hours. Real-time PCR was used to determine the mRNA level of macrophage phenotypic markers. (F) Phagocytic activities of mouse peritoneal macrophages were determined following co-treatment with PDTC and CM from TSP50-o/e cells for 24 hours. GAPDH was used as the internal control to check the efficiency of cDNA synthesis and PCR amplification. Data are shown as mean ± SD of three independent experiments. * p<0.05, **p<0.01.

We next tested whether NF-κB pathway is involved in this process, since the NF-κB pathway responds to stimulation by TNF-α and IL-1β [28]. Western blotting showed that following the treatment of CM of TSP50-o/e cells, phosphorylation of IκB kinase (IKK), IκBα and p65 Ser536 increased, and more p65 was translocated into the nucleus in dTHP-1 cells and mouse peritoneal macrophages (Fig 5A and S5A Fig), which suggested that the CM of TSP50-o/e cells activated the NF-κB pathway in macrophages. However, TNF-α shRNA-CM lost the ability to enhance IKK phosphorylation and p65 translocation in both human and mouse macrophages (Fig 5B and S5B Fig). Similar results were found in macrophages treated with IL-1β siRNA-CM (S5C Fig). In contrast, TNF-α and IL-1β increase the activities of the NF-κB pathway [28, 29]. These results suggested that TNF-α and IL-1β in the CM of TSP50-o/e cells might be responsible for the activation of the NF-κB signaling pathway in macrophages. We next examined whether the NF-κB signaling pathway contributed to the activation and polarization of macrophages induced by the CM of TSP50-o/e cells. We pretreated the macrophages with PDTC, a specific inhibitor of the NF-κB pathway before adding the CM of TSP50-o/e cells and observed that the induced IKK phosphorylation and p65 nuclear translocation were abolished, as expected (Fig 5C). Similarly, the secretion of IL-1β and induced expression of *CCL-1* and *IL-10* were also obviously inhibited by PDTC in dTHP-1 cells and mouse peritoneal macrophages (Fig 5D and 5E), whereas the expression of *CCL-5* was increased (S5C Fig). Additionally, PDTC treatment also reduced the phagocytic abilities of both murine and human macrophages (Fig 5F and S5D Fig). We also treated the macrophages with NAC, another inhibitor of the NF-κB pathway, before adding the CM of TSP50-o/e cells, and observed inhibition of IKK and IκB phosphorylation (S5E Fig). Taken together, these data demonstrated that the CM of TSP50-o/e cells activated and polarized macrophages via the NF-κB signaling pathway in a pro-inflammatory cytokine-dependent manner.

### 8. TSP50-o/e cells induced Inflammation, macrophage infiltration and M2b polarization in vivo

**Fig 6:**
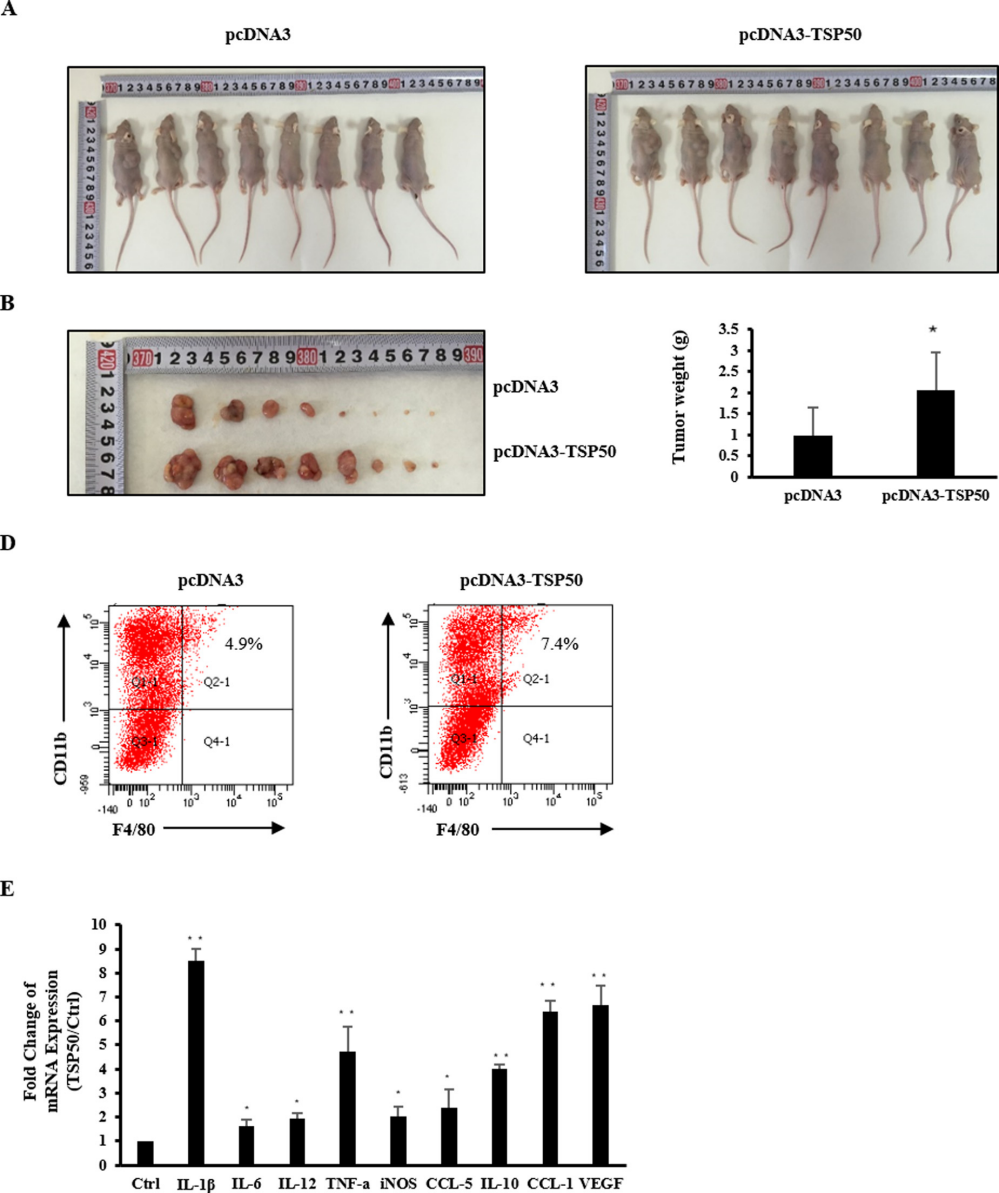
TSP50-o/e cells induced Inflammation, macrophage infiltration and M2b polarization in vivo. (A) Images of all the nude mice from each group (n = 8) at the end of the experiments. (B) Photographs of the dissected tumors from each group. (C) Tumor weight of each group. (D) Images representing the FACS data showing the quantification of CD11b and F4/80 macrophages infiltrated in TSP50-o/e tumors or the control tumors. The experiment was repeated three times with three to four mice in each group and similar results were obtained. (E) Quantitative PCR analysis of mRNA for M1- and M2-specific genes in the macrophages sorted from TSP50-o/e tumors or the control tumor. Data are shown as mean ± SD of three independent experiments. Statistical significance is assessed by a two sample t-test, where *denotes P<0.05, and ** denotes P<0.01.

To validate the effect of TSP50 expression in tumor cells on macrophages in vivo, we generated xenograft tumors in nude mice through subcutaneous injection of TSP50- o/e or control cells into the dorsal side of rear leg. We observed that the tumor size and tumor weight were dramatically increased in TSP50-o/e cell xenografts (Fig 6A–6C). To characterize the macrophages present in the tumors, macrophages (CD11b^+^, F4/80^+^) were sorted from TSP50-o/e or control tumors (Fig 6D). RNA from the sorted macrophages was extracted and used to determine the expression of M1- and M2b-specific genes. Real-time PCR analysis showed that the expressions of both M1 and M2b phenotypic markers were enhanced in TSP50-o/e tumors compared with control tumors. The expression of M2b markers (*IL-10*, *CCL-1* and *VEGF*) were increased to a greater extent than the expression of M1 markers (*iNOS*, *IL-12*) (Fig 6E). These in vivo data confirmed that overexpression of TSP50 in tumors affected the activities of macrophages in the tumor microenvironment and might contribute to tumor escape.

## Discussion

TSP50, as a protumoral factor, could initiate tumorigenesis and promote cell proliferation and colony formation, whereas knock-down of TSP50 induced apoptosis and repressed the proliferation of cancer cells [7, 8, 10]. Abnormal, high expression of TSP50 is consistently observed in many cancer tissues and is associated with poor prognosis [2, 3]. In this study, we identified the non-autonomous effect of TSP50 on the activities and polarization of macrophages, the key orchestrators of the tumor microenvironment, through paracrine secretion of cytokines. These results implied that TSP50 may facilitate tumor growth and development in vivo via multiple and diverse mechanisms.

Crosstalk between tumor cells and their microenvironment is pivotal for tumorigenesis and tumor progression. Tumors must manipulate their microenvironment to one that is at least growth permissive if not growth promoting [14]. This is probably because of the oncogenic factors or signaling pathways of the tumor cells that lead to secretion of molecules that alter the cellular composition and function of the microenvironment. For example, oncogenic MYC influences the tumor microenvironment, including suppressing the host immune responses, and MYC inactivation could contribute to tumor regression through the restoration of immune mechanisms [30, 31]. In this study, we observed that both primary macrophages and monocyte/macrophage cell lines were induced to produce a variety of cytokines and displayed enhanced phagocytic ability after exposure to the CM of TSP50-o/e cells. The cytokines produced by induced macrophages included both pro-inflammatory cytokines, such as IL-1β, IL-6 and TNF-α, and anti-inflammatory cytokines, such as IL-10, and TGF-β (Fig 1). Although these pro-inflammatory cytokines normally enhance the immune response and induce tumor cell death, they have paradoxical roles in the host defense against cancer. Over-production of these cytokines contributes to carcinogenesis in the early stage of tumor development [32]. IL-10 is considered as the most important anti-inflammatory cytokine and has a suppressive effect on the Th1 cell response. Therefore, IL-10 indeed associates with tumor-induced immune suppression and tolerance, and the presence of IL-10 in the microenvironment is positively associated with progression and development of cancer [33, 34]. However, IL-10 also increases the phagocytic activity of macrophages [35]. Accordingly, in this study we also found that the phagocytic index of macrophages was upregulated after exposure to the CM of TSP50-o/e cells or co-culture with TSP50-o/e cells (Fig 1).

Heterogeneity is one of the most important features of macrophages. Macrophages can be polarized into distinct phenotypes in different diseases and secrete different chemokines [19]. M1 macrophages secrete CCL-5, CXCL9, CXCL10 and CXCL5, promote the recruitment of Th1 and NK cells, and show tumoricidal activity [16, 25, 36]. In most tumors, the infiltrated macrophages are considered to be of the M2 phenotype (M2a, M2b and M2c), which provides an immunosuppressive microenvironment for tumor growth. M2a macrophages can promote the recruitment of eosinophils and basophils by secreting CCL24 and CCL17 [16], and can promote tumor cell migration [37]. M2b macrophages always secrete CCL1 and exert immune regulation. CCL-1 is specifically secreted from activated M2b macrophages and is essential for the maintenance of M2b properties [24]. Moreover, M2b macrophages are also considered potential targets of tissue repair in spinal cord injury and *Neisseria gonorrhoeae* infection [38, 39]. Finally, M2c macrophages secrete CCL16 and CCL18, which play an important role in immune suppression. It is reported that macrophages treated with the CM of MDA-MB231 cells were polarized into M2c subtype [40]. Here, we demonstrated that TSP50-o/e cells could mainly polarize surrounding macrophages into the M2b phenotype (CCL-1^high^, IL-10^high^, CCL-5^low^, IL-12^low^ and iNOS^low^) in vitro, and TSP50 might drive the tumor microenvironment to a suppressive state and facilitate tumor growth by inducing macrophages to polarize into an M2 phenotype.

TSP50 is a member of the peptidase S1 family of serine proteases and our previous study showed that TSP50 is mainly localized in the endoplasmic reticulum (ER) and cytoplasm membrane [41]. The extracellular secretion of TSP50 has hardly been detected, as observed in this study (Fig 3). Therefore, it is unlikely that TSP50 itself regulates the activities of macrophages directly. We found that production of several cytokines such as IL-1β, IL-10 and TNF-α were obviously induced from the TSP50-o/e cells. Among them, TNF-α and IL-1β were identified as the main factors responsible for the effect of the CM of TSP50-o/e cells on macrophages. Silencing TNF-α or IL-1β in TSP50-o/e cells abolished the TSP50-induced production of IL-1β, TNF-α and IL-10 (Fig 4). Subsequently, the CMs from TSP50-o/e cells with TNF-α or IL-1β silencing lost their effects on macrophages, such as the production of CCL-1 and IL-10, and reduced the phagocytic activities of the macrophages. Direct treatment of macrophages with TNF-α reproduced most of the effect of the CM of TSP50–o/e cells on macrophages, except IL-10 induction. IL-10 is one of the representative cytokines secreted by M2 macrophages and mediates the immunosuppressive effect. TNF-α, as a pro-inflammatory cytokine, is involved in M1, but not in M2, macrophage polarization. Therefore, TNF-α induced by TSP50 mediates most of the observed effect of TSP50 on macrophages; however, IL-10 expression in macrophages might be stimulated by other TSP50-induced cytokines, such as IL-10 itself in the CM of TSP50-o/e cells. Additional studies will be needed to clarify which cytokine plays a more important role in these processes and the mechanism by which TSP50 induces IL-10 synthesis in macrophages.

NF-κB is a central transducer of signals that cause inflammation and its activation is essential for macrophage recruitment and maturation, as well as the production of downstream pro-inflammatory cytokines and chemokines [42]. In addition, TNF-α and IL-1β are major upstream cytokines that can activate the NF-κB pathway. Here, we demonstrated that the CM of TSP50-o/e cells could significantly activate NF-κB pathway in macrophages (Fig 5). The activated NF-κB pathway was involved in mediating the effects of the CM from TSP50-o/e cells on macrophages, because pretreatment of macrophages with PDTC or NAC resulted in a reduction in cytokines production and phagocytosis induced by the CM of TSP50-o/e cells (Fig 5). However, we could not exclude the possibility that other signaling pathways might also contribute to the effects of TSP50 on macrophages. Therefore, we concluded that TSP50 elicits its effect on macrophages at least partly through inducing the release of TNF-α and IL-1β, and the subsequent activation of NF-kB pathway.

Accumulating evidence suggests that dynamic changes in the phenotypes of macrophages are actively involved in tumor initiation, progression and metastasis. Macrophages from different stages of a tumor vary [43]. M1 macrophages with TNF-α/IL-1β production and an active NF-κB pathway are central to tumor causal inflammation and play an important role in tumorigenesis. In established tumors, macrophages display the M2/trophic phenotype, with decreased TNF-α and repressed NF-κB pathway. In liver tumors, macrophages act in an M1-like manner at first, and are transformed into an M2-like phenotype during the development of the tumor [44]. The in vivo experiments showed that markers of the M1 and M2b phenotype were both increased, and the expression of M2b marker was increased to a greater extent. Based on the preliminary data in this study, we speculated that TSP50 might elicit its non-cell autonomous protumoral function at different stages of tumor development. It may promote tumorigenesis via activating pro-inflammatory macrophages, and after tumor initiation, TSP50 could maintain tumor growth through inducing M2 macrophage polarization to support an immunosuppressive environment. Moreover, TSP50 might also influence tumor invasion and metastasis by acting on macrophages in the tumor microenvironment. Therefore, further studies are necessary to fully investigate the effect of TSP50 on the tumor microenvironment.

In summary, the results obtained here demonstrated that oncogenic TSP50 expressed in the cells could activate surrounding macrophages and induce M2b polarization, partly through inducing TNF-α/IL-1β secretion, and NF-κB pathway activation. These phenomena imply that TSP50 might also regulate the tumor microenvironment to support tumor development. These findings provide an insight into the relationship between oncogenes and immune cells in the host microenvironment and provide a deeper understanding of the immune response in tumorigenesis. Moreover, this study also suggested that TSP50 might be a therapeutic target for cancer in the future.

## Supporting Information

S1 Fig CM of TSP50-o/e cells activated cytokines production and phagocytosis by macrophages. (A) Raw264.7 cells were cultured in medium containing 30% of CM from TSP50-o/e cells or control cells for 24h. Cytokine production in RAW264.7 cells was determined by real-time PCR. (B) Macrophages were exposed to CM from TSP50-o/e cells or control cells for 6h, 12h and 24h. IL-12 production in dTHP-1 cells (up) and mouse peritoneal macrophages (down) was determined by real-time PCR. (C, D) Phagocytosis of mouse peritoneal macrophages to cRBC was evaluated after 24 hours of CM treatment (C) or co-culture with TSP50-o/e cells or control cells (D). Mouse peritoneal macrophages phagocytizing cRBC were observed under a light microscope (left) and the calculated phagocytic index is shown (right). GAPDH was used as the internal control to check the efficiency of cDNA synthesis and PCR amplification. Representative results are from one of three independent experiments with similar results. Data are shown as mean ± SD of three independent experiments. * p<0.05, **p<0.01.
(TIF)

S2 Fig Knockdown of TNF-α in TSP50-o/e cells decreased macrophage activities. (A) TSP50-o/e cells were transfected with indicated shRNA plasmids for 24h, CM from TSP50-o/e cells or control cells was collected and subjected to ELISA to detect the secretion of TNF-α. (B) Macrophages were exposed to CM from TNF-α knockdown TSP50-o/e cells or control cells and collected at the given time points. Cytokine production in mouse peritoneal macrophages was determined by real-time PCR. (C) dTHP-1 cells were cultured with CM from TNF-α knockdown TSP50-o/e cells or control cells for 24h. The macrophages were collected and lysed, and the protein level of macrophage phenotypic markers were analyzed by western blotting. GAPDH was used as the internal control to check the efficiency of cDNA synthesis and PCR amplification. Data are shown as mean ± SD of three independent experiments. * p<0.05, **p<0.01.
(TIF)

S3 Fig Recombinant TNF-α induced macrophage activation and polarization. dTHP-1 cells and mouse peritoneal macrophages were treated with 40ng/mL of TNF-α or PBS for 24h. (A) The mRNA level of cytokines and macrophage phenotypic markers were analyzed by real-time PCR. (B) The protein levels of macrophage phenotypic markers were also determined by western blotting. (C) Phagocytosis activities of dTHP-1 cells (up) or mouse peritoneal macrophages (down) were determined after treatment with 40 ng/mL of TNF-α for 24 hours. Data are shown as mean ± SD of three independent experiments. * p<0.05, **p<0.01.
(TIF)

S4 Fig IL-1β in the CM of TSP50-o/e cells Affected the Activities of Macrophages. (A-B) TSP50-o/e cells were transfected with indicated siRNA plasmids. After 24h, the mRNA level of IL-1β (A) and cytokines (B) in these cells were analyzed by real-time PCR.(C-D) Mouse peritoneal macrophages were cultured with CM from IL-1β knock-down TSP50-o/e cells or control cells for 24h. The mRNA level of cytokines and macrophage phenotypic markers were determined by real-time PCR (C). The concentrations of phenotypic markers were measure using ELISA kits (D) GAPDH was used as the internal control to check the efficiency of cDNA synthesis and PCR amplification. Data are shown as mean ± SD of three independent experiments. * p<0.05, **p<0.01.
(TIF)

S5 Fig Activation of NF-κB pathway is related to the effects of TSP50-o/e CM on macrophages. (A) Macrophages were treated with CM from TSP50-o/e cells or control cells for 15min, 30min or 60min. The activation of the NF-κB pathway in mouse peritoneal macrophages was analyzed by western blotting. (B) dTHP-1 cells were treated with CM from TNF-α knock-down TSP50-o/e cells for 30min. The activation of the NF-κB pathway in dTHP-1 cells was then analyzed by western blotting.(C) dTHP-1 cells were treated with CM from IL-1β knockdown TSP50-o/e cells for 30min. The activation of the NF-κB pathway in dTHP-1 cells was then analyzed by western blotting.(D) Phagocytic activities of dTHP-1 cells were determined following co-treatment with PDTC and CM from TSP50-o/e cells for 24 hours.(E) dTHP-1 cells were pretreated with 15mM NAC for 1 hour and then the culture medium were replaced with fresh medium containing 30% of CM from TSP50-o/e cells or control cells. After 30-min of incubation, the activation of the NF-κB pathway was analyzed by western blotting.Data are shown as mean ± SD of three independent experiments. * p<0.05, **p<0.01.
(TIF)
