# Supplementary figures and images for: Protumoral TSP50 Regulates Macrophage Activities and Polarization via Production of TNF-α and IL-1β, and Activation of the NF-κB Signaling Pathway
Source: PLoS One. 2015 Dec 18;10(12):e0145095. doi: 10.1371/journal.pone.0145095 (PMC4684331; doi:10.1371/journal.pone.0145095)

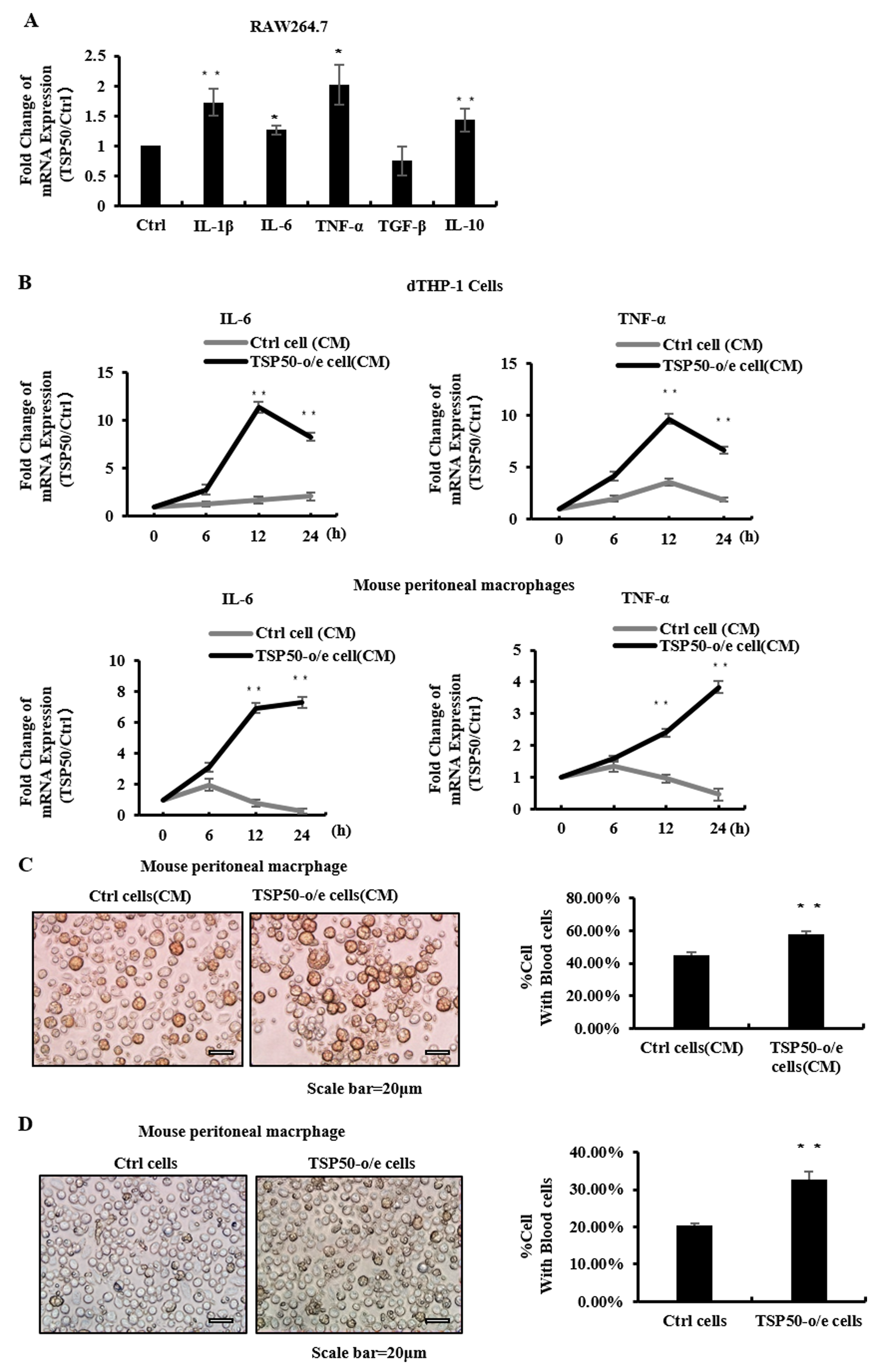

Supplement: S1 Fig — (A) Raw264.7 cells were cultured in medium containing 30% of CM from TSP50-o/e cells or control cells for 24h. Cytokine production in RAW264.7 cells was determined by real-time PCR. (B) Macrophages were exposed to CM from TSP50-o/e cells or control cells for 6h, 12h and 24h. IL-12 production in dTHP-1 cells (up) and mouse peritoneal macrophages (down) was determined by real-time PCR. (C, D) Phagocytosis of mouse peritoneal macrophages to cRBC was evaluated after 24 hours of CM treatment (C) or co-culture with TSP50-o/e cells or control cells (D). Mouse peritoneal macrophages phagocytizing cRBC were observed under a light microscope (left) and the calculated phagocytic index is shown (right). GAPDH was used as the internal control to check the efficiency of cDNA synthesis and PCR amplification. Representative results are from one of three independent experiments with similar results. Data are shown as mean ± SD of three independent experiments. * p<0.05, **p<0.01. (TIF) [file pone.0145095.s001.tif]

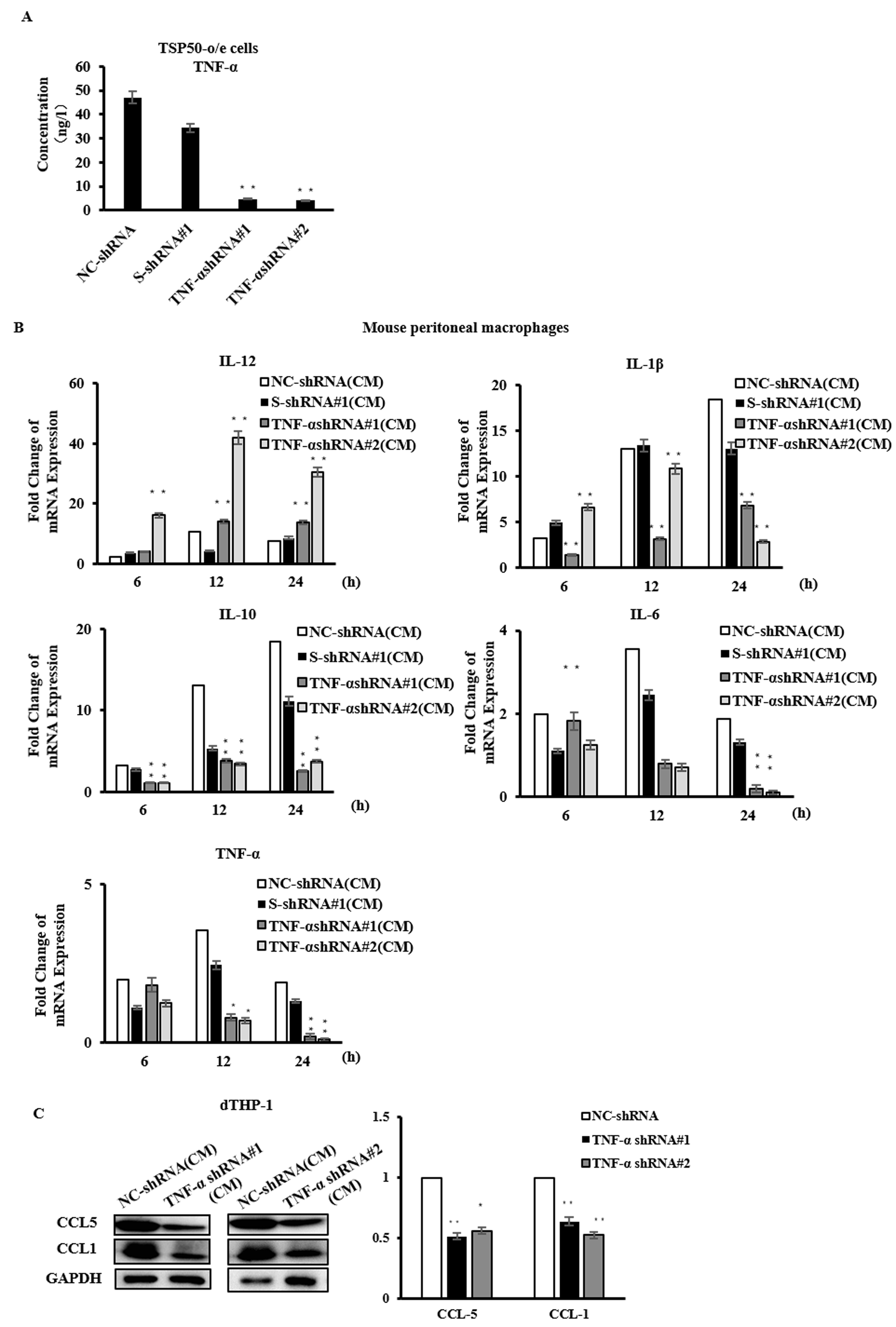

Supplement: S2 Fig — (A) TSP50-o/e cells were transfected with indicated shRNA plasmids for 24h, CM from TSP50-o/e cells or control cells was collected and subjected to ELISA to detect the secretion of TNF-α. (B) Macrophages were exposed to CM from TNF-α knockdown TSP50-o/e cells or control cells and collected at the given time points. Cytokine production in mouse peritoneal macrophages was determined by real-time PCR. (C) dTHP-1 cells were cultured with CM from TNF-α knockdown TSP50-o/e cells or control cells for 24h. The macrophages were collected and lysed, and the protein level of macrophage phenotypic markers were analyzed by western blotting. GAPDH was used as the internal control to check the efficiency of cDNA synthesis and PCR amplification. Data are shown as mean ± SD of three independent experiments. * p<0.05, **p<0.01. (TIF) [file pone.0145095.s002.tif]

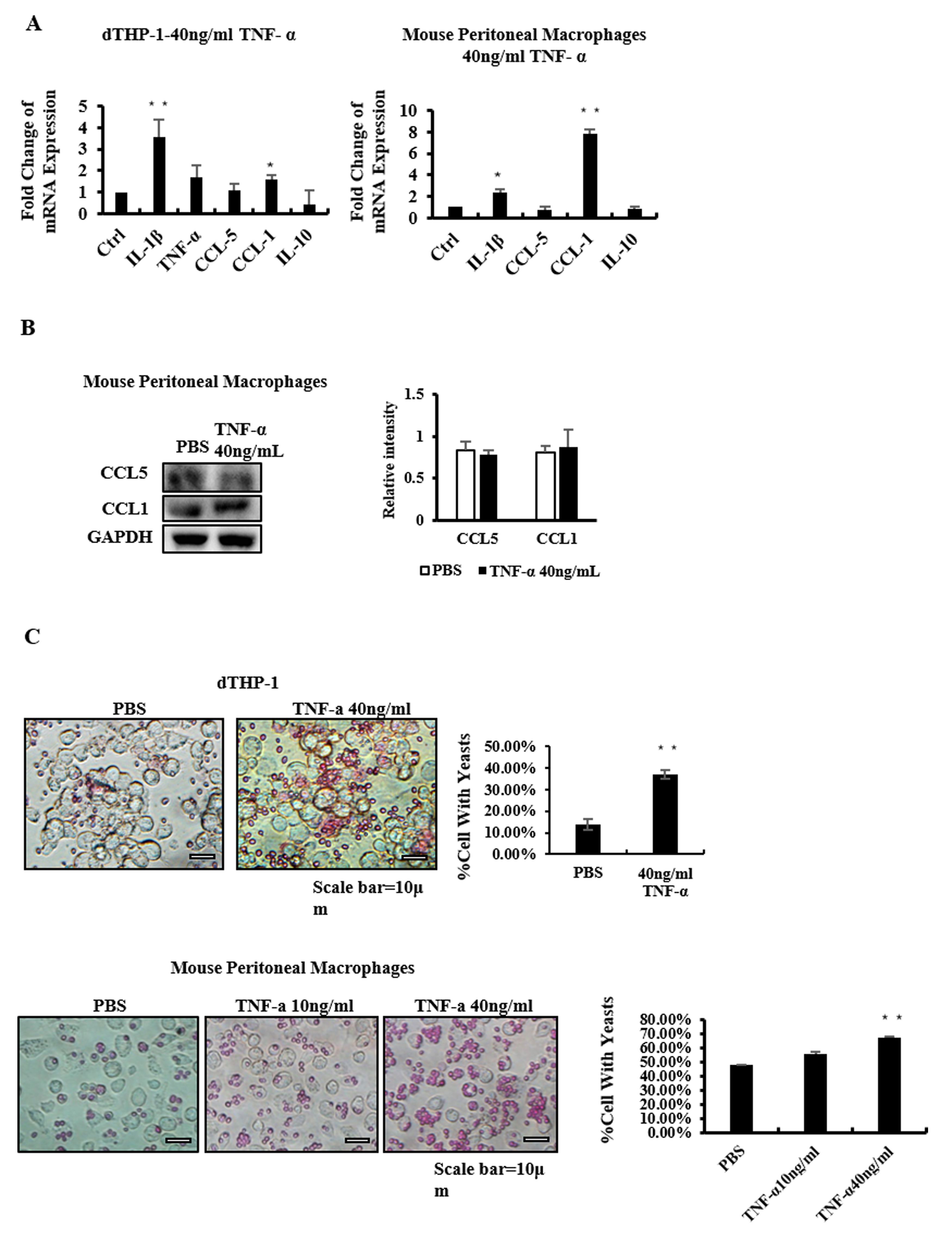

Supplement: S3 Fig — dTHP-1 cells and mouse peritoneal macrophages were treated with 40ng/mL of TNF-α or PBS for 24h. (A) The mRNA level of cytokines and macrophage phenotypic markers were analyzed by real-time PCR. (B) The protein levels of macrophage phenotypic markers were also determined by western blotting. (C) Phagocytosis activities of dTHP-1 cells (up) or mouse peritoneal macrophages (down) were determined after treatment with 40 ng/mL of TNF-α for 24 hours. Data are shown as mean ± SD of three independent experiments. * p<0.05, **p<0.01. (TIF) [file pone.0145095.s003.tif]

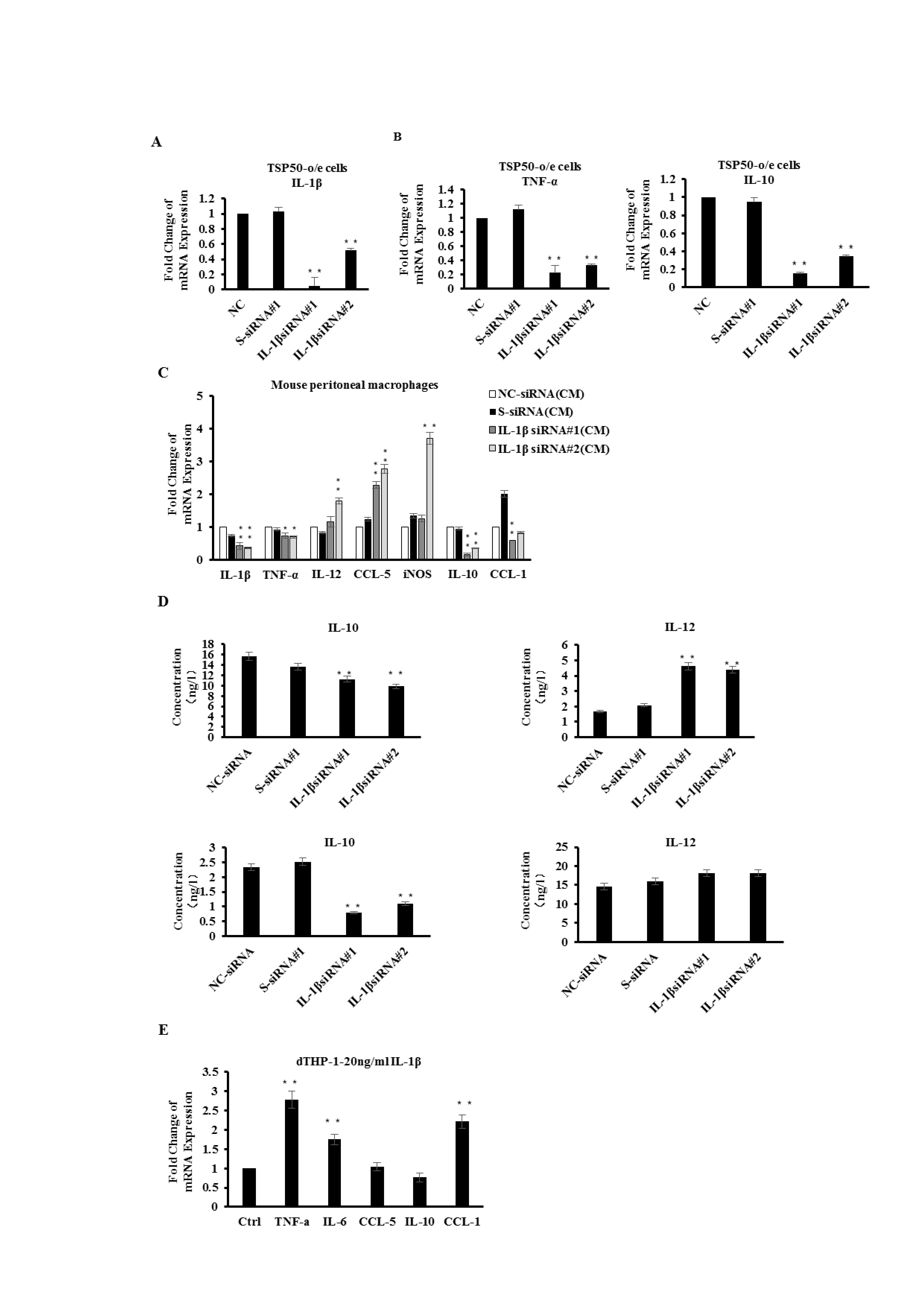

Supplement: S4 Fig — (A-B) TSP50-o/e cells were transfected with indicated siRNA plasmids. After 24h, the mRNA level of IL-1β (A) and cytokines (B) in these cells were analyzed by real-time PCR.(C-D) Mouse peritoneal macrophages were cultured with CM from IL-1β knock-down TSP50-o/e cells or control cells for 24h. The mRNA level of cytokines and macrophage phenotypic markers were determined by real-time PCR (C). The concentrations of phenotypic markers were measure using ELISA kits (D) GAPDH was used as the internal control to check the efficiency of cDNA synthesis and PCR amplification. Data are shown as mean ± SD of three independent experiments. * p<0.05, **p<0.01. (TIF) [file pone.0145095.s004.tif]

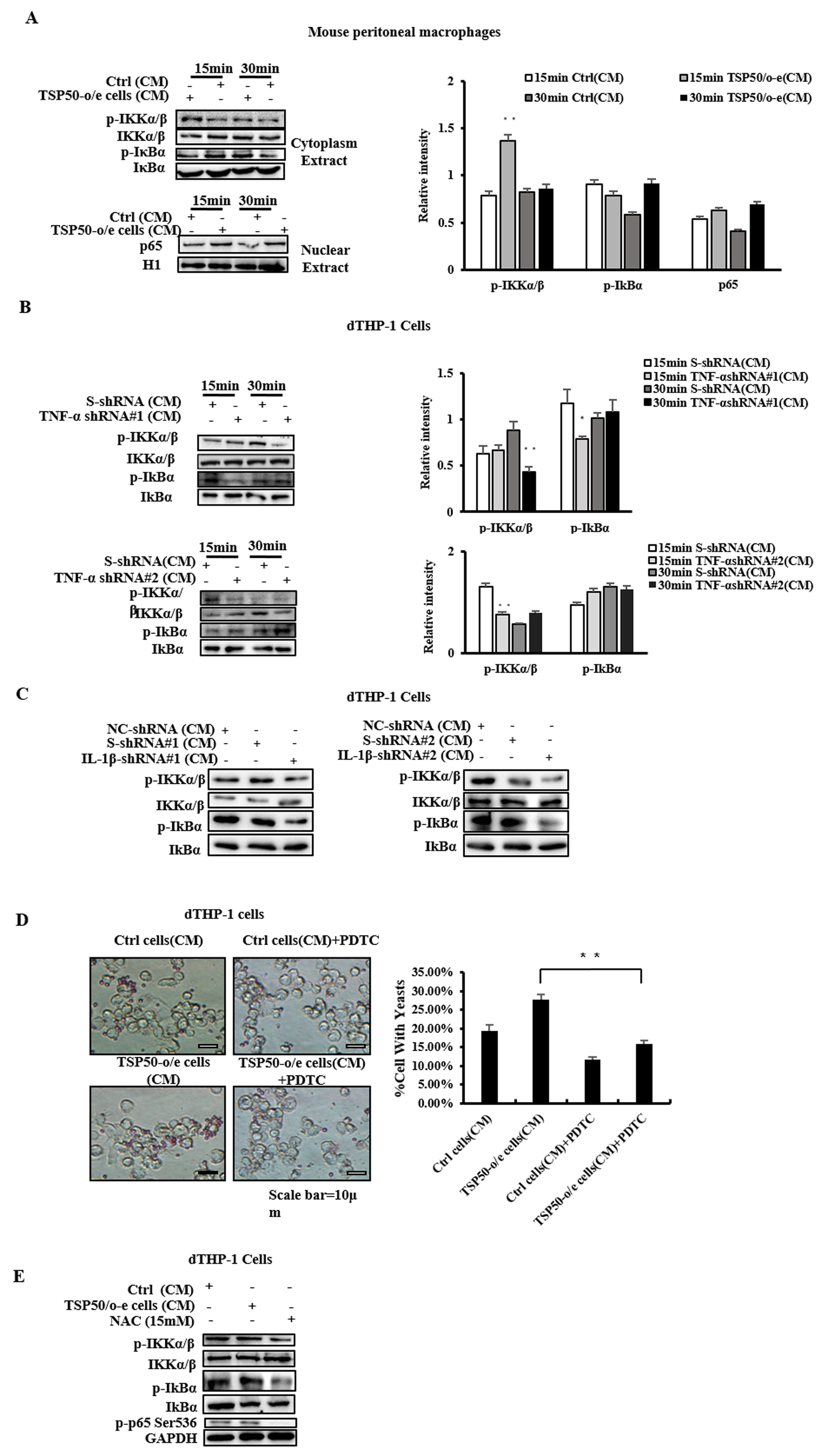

Supplement: S5 Fig — (A) Macrophages were treated with CM from TSP50-o/e cells or control cells for 15min, 30min or 60min. The activation of the NF-κB pathway in mouse peritoneal macrophages was analyzed by western blotting. (B) dTHP-1 cells were treated with CM from TNF-α knock-down TSP50-o/e cells for 30min. The activation of the NF-κB pathway in dTHP-1 cells was then analyzed by western blotting.(C) dTHP-1 cells were treated with CM from IL-1β knockdown TSP50-o/e cells for 30min. The activation of the NF-κB pathway in dTHP-1 cells was then analyzed by western blotting.(D) Phagocytic activities of dTHP-1 cells were determined following co-treatment with PDTC and CM from TSP50-o/e cells for 24 hours.(E) dTHP-1 cells were pretreated with 15mM NAC for 1 hour and then the culture medium were replaced with fresh medium containing 30% of CM from TSP50-o/e cells or control cells. After 30-min of incubation, the activation of the NF-κB pathway was analyzed by western blotting.Data are shown as mean ± SD of three independent experiments. * p<0.05, **p<0.01. (TIF) [file pone.0145095.s005.tif]
